# Nursing staffs self-perceived outcome from a rehabilitation 24/7 educational programme – a mixed-methods study in stroke care

**DOI:** 10.1186/s12912-018-0285-z

**Published:** 2018-04-27

**Authors:** M. I. Loft, B. A. Esbensen, K. Kirk, L. Pedersen, B. Martinsen, H. Iversen, L. L. Mathiesen, I. Poulsen

**Affiliations:** 1 Department of Neurology, Rigshospitalet, Nordre Ringvej 57, 2600 Glostrup, Denmark; 2 Institute of Public Health, Department of Nursing Science, Aarhus University, Aarhus, Denmark; 3 Copenhagen Centre for Arthritis Research (COPECARE), Centre for Rheumatology and Spine Diseases VRR, Head and Orthopaedics Centre, Rigshospitalet, Glostrup, Denmark; 4 Falcuty of Health and Medical Sciences, Department of Clinical Medicine, University of Copenhagen, Copenhagen, Denmark; 5 Partner PAR3(consulting firm), Copenhagen, Denmark; 6 Research Unit on Brain Injury Rehabilitation Copenhagen (RuBRIC), Clinic of Neurorehabilitaion, TBI unit Rigshospitalet, Glostrup, Denmark

**Keywords:** Behaviour change, Complex intervention, Feasibility, Stroke, Rehabilitation

## Abstract

**Background:**

During the past two decades, attempts have been made to describe nurses’ contributions to the rehabilitation of inpatients following stroke. There is currently a lack of interventions that integrate the diversity of nurses’ role and functions in stroke rehabilitation and explore their effect on patient outcomes. Using a systematic evidence- and theory-based design, we developed an educational programme, Rehabilitation 24/7, for nursing staff working in stroke rehabilitation aiming at two target behaviours; working systematically with a rehabilitative approach in all aspects of patient care and working deliberately and systematically with patients’ goals. The aim of this study was to assess nursing staff members’ self-perceived outcome related to their capability, opportunity and motivation to work with a rehabilitative approach after participating in the stroke Rehabilitation 24/7 educational programme.

**Methods:**

A convergent mixed-method design was applied consisting of a survey and semi-structured interviews. Data collection was undertaken between February and June 2016. Data from the questionnaires (*N* = 33) distributed before and after the intervention were analysed using descriptive statistics and Wilcoxon sign rank test. The interviews (*N* = 10) were analysed using deductive content analysis. After analysing questionnaires and interviews separately, the results were merged in a side by side comparison presented in the discussion.

**Results:**

The results from both the quantitative and qualitative analyses indicate that the educational programme shaped the target behaviours that we aimed to change by addressing the nursing staff’s capability, opportunity and motivation and hence could strengthen the nursing staff’s contribution to inpatient stroke rehabilitation. A number of behaviours changed significantly, and the qualitative results indicated that the staff experienced increased focus on their role and functions in rehabilitation practice.

**Conclusion:**

Our study provides an understanding of the outcome of the Rehabilitation 24/7 educational programme on nursing staff’s behaviours. A mixed-methods approach provided extended knowledge of the changes in the nursing staff members’ self-percived behaviours after the intervention. These changes suggest that educating the nursing staff on rehabilitation using the Rehabilitation 24/7 programme strengthened their knowledge and beliefs about rehabilitation, goal-setting as well as their role and functions**.**

## Background

Every year, 15 million people worldwide are affected by stroke. Stroke has physical, mental and social consequences for patients, with many patients subsequently needing rehabilitation. We know that both early initiation and the intensity of patients’ rehabilitation affect functional outcomes [1–3]. However, patients experience time wastage during inpatient rehabilitation that could profitably have been used for active rehabilitation [4, 5].

The term ‘rehabilitation’ refers to a targeted and time-delimited process that involves collaboration between different professionals, the patient and the relatives. This process should be undertaken with defined, meaningful goals that are formulated together with the patient [6, 7]. In this understanding of rehabilitation, a holistic approach to disease is adopted, where physical, psychological and social consequences are taken into account. The goals for rehabilitation are thus not merely the absence of disease and symptoms, but relate to the patient getting to live independently and participate in meaningful social activities with the highest possible self-perceived quality of life. Patients affected by stroke require specialized skills from staff as they often have motor, cognitive, speech and behavioural sequelae that complicate their involvement in the rehabilitation process [6].

Nurses play an important role in stroke rehabilitation and are a natural member of the interdisciplinary rehabilitation team [8, 9]. However, their role and functions appear to be therapeutically unclear and the nursing staff in a stroke rehabilitation unit often fail to fully incorporate rehabilitation practices into their daily routines[RW.ERROR - Unable to find reference:562]. Previous, mainly descriptive, research on nurses’ role and functions in stroke rehabilitation gave rise to a call for interventions that focused on strengthening their contributions [8–10].

Research indicates that nursing interventions are often underdeveloped, and this likely explains why they have difficulties showing effect [11, 12]. Furthermore, research findings are poorly integrated in practice [11, 13]. It is therefore important to develop theory- and evidence-based interventions where also the implementation strategy is incorporated from the beginning [13–15]. An intervention for stroke rehabilitation that could strengthen the nursing staff’s contributions is a complex intervention that largely centres on behavioural change. We therefore chose to develop an evidence- and theory-based intervention guided by the British Medical Research Council’s (MRC) framework for complex interventions [15] to ensure a systematic and evidence based development and Michie’s Behaviour Change Wheel (BCW) [14] as the intervention would address behaviour change [16]. Furthermore, we addressed implementation from the outset by developing a strategy following the steps recommended by Grol and Wensing [13]. Trials of complex interventions are by nature complex in their design and overall process; thus, feasibility studies are valuable for informing trials that relate to clinical, procedural or methodological issues before setting up an effect study [11]. We have previously investigated the educational programme for its feasibility and acceptability (under review). As a next step, we in this study investigate if there is any self-perceived changes in the nursing staff members’ behaviours related to capability, opportunity and motivation (COM-B factors) [14]. To our knowledge, this is the first study to investigate whether an intervention obtained on the basis of BCW benefits the COM-B factors or whether something should be changed before a larger effect study is carried out.

### Aim

The aim of this convergent mixed-methods study was to asses nursing staff members’ self-perceived outcome on their capability, opportunity and motivation to work with a rehabilitative approach after participating in the stroke Rehabilitation 24/7 educational programme.

## Methods

### Design

**Fig. 1:**
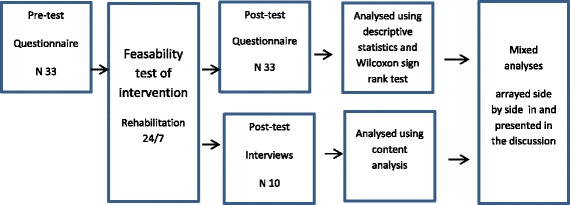
Study overview

A convergent mixed-method design was applied that included a questionnaire to obtain quantitative data and semi-structured interviews to obtain qualitative data. The rationale for choosing a mixed-methods design was the recognition of a need for different methods that, in combination, can give a better understanding of the complex contextual environment of healthcare [11, 17, 18]. For a study overview, see Fig. 1.

### **S**etting and intervention

The educational programme named “Rehabilitation 24/7” was designed to strengthen the nursing staff’s rehabilitative practices. The intervention addressed key barriers identified within the COM-B model with the purpose of achieving behavioural change in the nursing staff [16]. The specific goals of the intervention for the nursing staff were as follows:

- To work systematically with a rehabilitative approach in all aspects of patient care
- To work deliberately and systematically with patients’ goals.

The intervention was designed as an educational programme that comprised three workshops lasting 3 h each, interspersed with work-in-practice. Over the course of a 3-month period in the spring of 2016, all registered nurses (RNs) (*n* = 19) and all nurse assistants (NAs) (*n* = 18) in a 15-bed acute stroke unit were enrolled in the educational programme. Participation was mandatory, except for substitute staff. Together with the first and last author of this paper, the two charge nurses of the unit and three nursing staff members were involved in designing and planning the intervention and took an active role in training. In developing and modelling the intervention, two professional advisers were also involved, a Master of Science (MSc) in Economics and a Master in Organisational Psychology. These two professional advisers contributed with knowledge of and experience in patient involvement, process improvement and change management. Moreover, they had insider perspectives as a former patient and as a relative, respectively. The intervention was developed based on existing relevant research and empirical research undertaken by the developers of the intervention (interviews and field observations in the chosen stroke unit) [16, 19, 20]. In the early development stages, field observations were also conducted in another rehabilitation inpatient setting to broaden perspectives and later to validate the findings. Each component in the intervention was furthermore based on theory and evidence hence for instance the didactic considerations were drawing on educational theory [21].

**Fig. 2:**
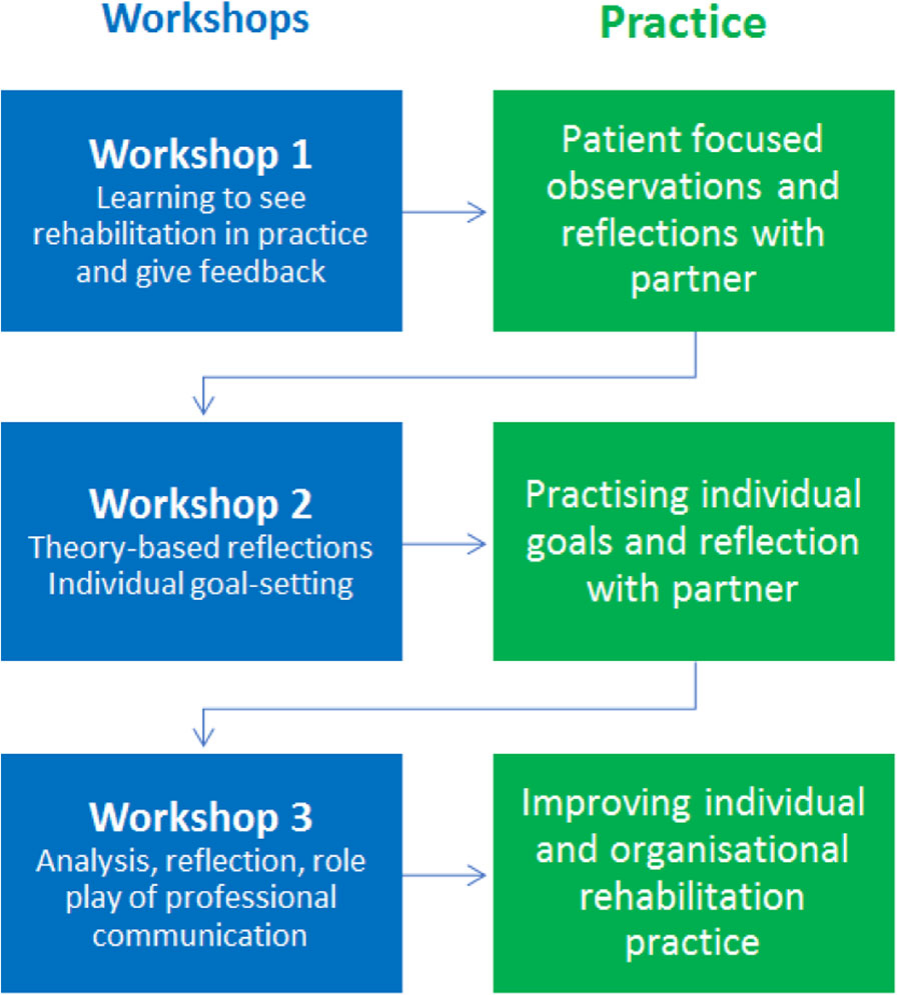
Rehabilitation 24/7 educational programme flowchart

The main elements of the educational programme outlined in Fig. 2 were as follows:

1. Improving the participants’ theoretical understanding of how nursing contributes to patients’ rehabilitation. Every workshop comprised theoretical input, including rehabilitation theory and history, theory related to nursing role and functions, patient narratives, and evidence related to goal-setting.
2. Presenting and providing training on tools for improving rehabilitation tasks. In workshop 1, the focus was on learning to give peer-to-peer feedback and performing patient-centred observations, which should be performed 2 to 3 h in the participants’ own practice. Guidelines were handed out for both of these elements. In workshop 2, the participants’ own experiences that derived from the observations were used to reflect on theoretical aspects of the nursing role.
3. Effecting change in practice. Each participant identified own areas for further development and was asked to formulate individual goals on which to work. The charge nurses were involved in developing the educational programme, and participated as well. This was done to increase ‘ownership’ of the changes at all levels, as well as ensure that the programme was designed to fit the particular needs of the stroke rehabilitation unit.

### Participants

All RNs and NAs participating in the educational programme were asked to answer the questionnaire immediately before and after the 7-week educational programme was completed.

For the semi-structured interviews, RNs and NAs were selected to obtain a purposive sample to ensure breadth and variety in perspectives [22]. Thus, RNs and NAs with differing degrees of seniority and ages were selected. The principle of data saturation, defined as the point at which no additional new information was obtained, guided sampling. After 10 interviews, the interviewer (BAE) and the first author (MIL) agreed that saturation had been reached, and data collection ceased.

For characteristics of the sample for the questionnaire and interviews, see Table 1.

**Table 1:** Sample characteristics for questionnaire and semi-structured interviews

Characteristics	N Questionnaire	Percentage (%) Questionnaire	N Interviews
Professional group			
Registered nurse Nurse assistant	17 16	51.5 48.5	6 4
Sex			
Male Female	2 31	6 94	1 9
Education (years since graduation)			
< 2 years 2–5 years > 5 years	7 1 25	21 3 76	2 0 8
Supplementary education			
Yes No	12 21	36 64	3 7
Current employment			
< 2 years 2–5 years > 5 years	13 6 14	40 18 42	3 1 6

Numbers answering questionnaire: 33 out of 37 participants

### Data collection

#### Questionnaire

The questionnaire was inspired by the COM-B Self-Evaluation Questionnaire and the COM-B Behavioural Diagnosis Form [14]. It was intended to examine self-perceived outcomes before and after the educational programme that related to the COM-B factors (capability [C], opportunity [O] and motivation [M]) [14] and patient involvement. The questionnaire was completed electronically before and after the educational programme. After the programme, the questionnaire was expanded with an additional 11 questions (see Table 3) addressing how the participants assessed their professional knowledge and skills after participating in the intervention. The questionnaire was reviewed for its comprehensibility and face validity with a convenience sample (*n* = 5) prior to being administered. The questionnaire used a 4-point Likert scale ranging from very bad to very good and totally disagree to totally agree (Table 4).

#### Interviews

The interviews were conducted using a semi-structured interview guide [22] that explored participants’ perceptions of the relevance of the educational programme and its learning outcomes. The interviews were also aimed at uncovering areas that related to RNs’ and NAs’ daily practice, indicating possible changes in the identified COM-B factors. All interviews were conducted in an outlying office at the hospital and lasted between 37 and 50 min (mean duration 45 min). The interviewer was unknown to the participants and had not been directly involved in the development or delivery of the educational programme. The interviews were digitally recorded and transcribed verbatim.

### Analyses

The questionnaires were analysed using descriptive statistics and Wilcoxon sign rank test using IBM SPSS Statistics [23].

The qualitative interview data were analysed using deductive content analysis as described by Elo and Kyngäs [24] as we aimed at investigating already predefined categories. COM-B factors were employed to develop a structured categorisation matrix [24]. This matrix was used as a lens for the analysis. The theme of the interviews was chosen to facilitate answering the research questions about the nursing staff members’ capability, opportunity and motivation in relation to their daily work in the stroke unit. Changes in the nursing staff members’ perceptions of their competence were analysed, coded and compared with the COM-B factors. The development of the intervention also involved identifying barriers which suggested the need to make changes to the COM-B factors, and this information constituted the basis for what we looked for in relation to each COM-B factor (Table 2). The material was transcribed verbatim and NVivo® software (QSR International Pty Ltd., Victoria, Australia) was used to provide an overview and facilitate a systematic approach to analysing the material. Three members of the research team (first, second and last author) independently read the transcripts multiple times to become familiar with the data and acquire an overview of the text. Then, the transcripts were reviewed for content. Text corresponding to the matrix categories was coded and transferred to these categories. Any coding inconsistencies among the researchers were discussed until consensus was reached. A description of each category was then developed.

**Table 2:** COM-B factors to look for in the analysis

COM-B factors:	Identified as:
Physical Capability	Having the physical skills to work towards the patient’s goals; having the physical skills to work systematically with a rehabilitative approach
Psychological Capability	Understanding nursing roles and functions in the rehabilitation process; understanding the concept of rehabilitation; knowing and understanding the reason for working with goals in rehabilitation; knowing how to collaborate with the patient; knowing how to collaborate with the interdisciplinary collaborators.
Physical Opportunity	Having the opportunity to document goal-setting, progress, etc.; having time, resources and the physical space to work systematically with a rehabilitative approach
Social Opportunity	Fostering a culture in which the nursing staff can work effectively and take co-responsibility for achieving the patient’s goals; observing senior nurses working deliberately and systematically with the patient’s goals in mind; having social and cultural norms that strengthen respect for nursing roles and functions; appreciating the value of a rehabilitative approach
Automatic Motivation	Following established routines and habits for working deliberately and systematically towards the patient’s goals and with a rehabilitative approach
Reflective Motivation	Having a strong professional identity; believing that it is possible to integrate rehabilitation principles into daily care; believing that a rehabilitative approach is important; believing that a systematic rehabilitative approach will improve the patient’s outcome

In the mixed analysis, results from the two individual analyses were brought together in a discussion where they are arrayed side by side this is in accordance with the methods as presented by Creswell [17, 18].

## Results

### Quantitative results

#### Self-perceived outcome

The nursing staff assessed the overall outcome of Rehabilitation 24/7 as having had a positive influence on both their professional identity and competencies (Table 3). Regarding the two target behaviours, 71% answered that they agreed or totally agreed feeling stronger in relation to working deliberately with the patient’s goals as part of their daily care; and 74.2% answered that they felt stronger working with a rehabilitative approach. This was also reflected in the result that 74.2% agreed or totally agreed about becoming aware of the possibilities of affecting rehabilitation within the profession (Table 3). When asked about the effect of the feedback element of the educational programme, 80.6% and 87.1% agreed or totally agreed, respectively, that they had improved as a result of giving feedback to or receiving feedback from their colleagues.

**Table 3:** Nursing staffs assessment of self-perceived outcome after participating in the educational programme

%	Totally agree	Agree	Disagree	Totally disagree
I feel more secure in my professional role and function	22.6	61.3	16.1	0.0
I feel more competent involving the patient in my work	16.1	67.8	16.1	0.0
I feel stronger about working deliberately with patients’ goals in daily care	12.9	58.1	29.0	0.0
I feel stronger working with a rehabilitative approach	25.8	48.4	25.8	0.0
I become aware of the possibilities within my profession to affect the patient’s rehabilitation	12.9	61.3	25.8	0.0
I improved at giving feedback to my colleagues	16.1	64.5	19.4	0.0
I improved at receiving feedback from my colleagues	22.6	64.5	12.9	0.0
I feel more competent at collaborating with the interdisciplinary team	19.4	67.7	12.9	0.0
I feel more competent at communicating my professional contribution to the interdisciplinary team	9.8	71.0	19.4	0.0
I feel more competent at communicating my professional contribution to the patient	16.1	64.5	19.4	0.0
I feel more competent at communicating my professional contribution to patients’ relatives	16.1	64.5	19.4	0.0

Among the participants 80.8% agreed or totally agreed that they had become more competent in relation to communicate their professional contribution to the interdisciplinary team, patients and relatives (Table 3).

#### Assessing own skills

When looking at the COM-B factors assessed before and after the educational programme, we found that the nursing staff assessed their skills in collaborating with doctors as having increased after the intervention (*p* = 0.038). There were no changes in skills involved in collaborating with colleagues within the nursing staff group. However, descriptive statistics showed a statistically near significant (*p* = 0.059) positive change in monodisciplinary collaboration. There were no reported changes in relation to skills used to involve patients in daily work. Skills that related to giving feedback increased significantly (*p* = 0.017), but no change was found with respect to receiving feedback (Table 4).

**Table 4:** Nursing staffs self-perceived outcome before and after participating in the educational programme

	Question		Before %				After %				Wilcoxon signed rank test	*P*-value
	How do you assess your skills?		Very good	Good	Bad	Very bad	Very good	Good	Bad	Very bad	z	*P*
1	To work patient involving		36	64	–	–	39	61	–	–	0.000	1.0
2	To work with the patients goals in daily care		15	79	–	–	23	74	3	–	−0.966	0.334
3	Focusing on rehabilitative principles in daily care		15	85	–	–	26	74	–	–	− 1.732	0.083
4	Collaborating with mono professional colleagues		30	70	–	–	45	55	–	–	−1.890	0.059
5	Collaborating with nursing staff colleagues		39	61	–	–	35	61	–	–	−0.832	0.405
6	Collaborating overall in the rehabilitation team		21	70	9	–	39	55	6	–	−1.371	0.170
7	Collaborating with therapists		18	70	12	–	39	55	6	–	−1.529	0.126
8	**Collaborating with the doctor**		18	70	12	–	23	74	3	–	− 2.070	**0.038**
9	Learning from colleagues on the interdisciplinary team		18	76	6	–	23	77	–	–	−1.613	0.107
10	**Giving feedback on colleagues’ work**		6	67	27	–	26	61	13	–	−2.379	**0.017**
11	Receiving feedback from colleagues		6	76	18	–	13	77	10	–	−1.333	0.182
	Rate your level of agreement or disagreement with the following statements regarding your competencies in the care and rehabilitation of patients admitted for rehabilitation after a stroke.	Totally agree	agree	dis -agree	Totally dis-agree	Totally agree	agree	dis -agree	Totally disagree		z	P
12	**I know how to work with a rehabilitative approach**	25	72	3	–	52	48	0	–		−2.496	**0.013**
13	**I know what patient involvement in rehabilitation means**	34	63	3	–	55	45	0	–		−2.111	**0.035**
14	I know how to motivate the patient to participate in their rehabilitation	31	66	3	–	45	55	0	–		−1.155	0.248
15	**I am sure of how to work with patients towards their goals for rehabilitation**	22	69	9	–	48	52	–	–		−2.653	**0.008**
16	**I am competent in performing the tasks I think are expected of me as a nurse/nurse assistant in a stroke rehabilitation unit**	31	63	6	–	52	48	–	–		−2.179	**0.029**
	Indicate your level of agreement or disagreement with the following statements regarding your role and function as a nurse/nurse assistant in the care and rehabilitation of patients admitted for rehabilitation after a stroke.											
17	**I know what is expected of me as an RN/NA in a rehabilitation unit**	19	71	10	–	47	53	–	–		−2.486	**0.013**
18	I know what patients expect of me	23	65	13	–	23	63	13	–		−0.543	0.587
19	I know what patients’ relatives expect of me	23	55	23	–	17	60	20	3		−1.273	0.203
20	I know what my colleagues in the care group expect of me	23	65	13	–	27	73	–	–		−1.473	0.141
21	I know what the doctor expects of me	16	65	19	–	17	77	7	–		−0.812	0.,417
22	I know what the therapist expects of me	16	68	16	–	17	77	7	–		−0.933	0.351
23	I estimate that I am so strong in my professional function that I can clearly communicate it to patients	29	52	16	3	33	63	3	–		−1.789	0.074
24	**I am so strong in my professional function that I can communicate it clearly to my interdisciplinary colleagues**	29	48	23	–	37	60	3	–		−1.979	**0.048**
25	I am competent to work with the other staff in the patient’s rehabilitation	35	58	6	–	47	50	3	–		−0.237	0.083
26	I know where my professional function differs from others in the interdisciplinary team	32	55	13	–	43	53	3	–		−0.722	0.470
27	I am happy with my work	42	48	10	–	47	43	–	–		−0.690	0.490
28	I am proud of my professional group’s contribution to patients’ rehabilitation	35	58	6	–	50	47	–	–		−1.732	0.083
29	I asses that my professional group has an untapped potential to contribute to the rehabilitation of patients admitted after a stroke	32	61	7	–	40	50	10	–		−0.087	0.931
	Indicate your level of agreement or disagreement with the following statements regarding how you work in the unit in terms of patient involvement.											
30	Within my professional group, we always know who is responsible for a patient	16	49	32	3	20	53	23	3		−0.549	0.583
31	It is in our department’s culture to involve patients throughout their rehabilitation	36	55	10	–	27	67	6	–		−0.749	0.454
32	We are good at translating the individual’s experiences/wishes into solutions that suit the patient	7	74	19	–	23	60	17	–		−1.027	0.305
33	Our division of labour between professional groups allows the patients to be involved	10	71	19	–	17	67	16	–		−0.104	0.917
34	In the department we are continuously discussing what is actually meant by involving patients	6	52	32	10	17	37	46	–		−0.514	0.607
35	The way we work together in the interdisciplinary team gives the patient a coherent experience of the rehabilitation course/pathway	13	74	10	3	17	70	13	–		−0.426	0.670
36	We have the resources to be able to involve the patients as a permanent practice	10	36	48	6	10	50	40	–		−1.079	0.281
37	The way the stroke unit works as an organisation supports patient involvement in the rehabilitation	13	64	23	–	17	60	23	–		−0.484	0.628
38	**We have organised the work so that the patients’ views and resources come into play**	23	64	13	–	10	70	20	–		−2.271	**0.023**
39	The way our work is organised enables us to have a holistic view of the patient	13	52	35	–	10	60	30	–		−0.302	0.763
40	In our department, we prioritise the contact person system	6	42	42	10	10	33	47	10		0.000	1.0
41	In our department, we prioritise the patient to experience continuity in contact with the nursing staff	19	52	23	6	7	63	30	–		−0.179	0.858
	Rate your level of agreement or disagreement with the following statements related to what you think you need in order to be even better at working with a rehabilitative approach.											
42	**I need to know more about why it is important to work using a rehabilitative approach**	10	45	42	3	3	20	57	20		−2.216	**0.027**
43	**I need education to know more about how to work with a rehabilitative approach**	13	58	29	–	3	27	60	10		−3.063	**0.002**
44	**I need more practical skills**	6	52	39	3	6	27	57	10		−2.166	**0.030**
45	I need more mental skills	6	58	36	–	3	50	40	7		−1.054	0.292
46	I need more time	42	39	19	–	43	37	20	–		−0.302	0.763
47	I need a different structure in the unit	6	62	29	3	13	57	27	3		−0.091	0.927
48	I need more colleagues in the nursing staff group	39	45	16	–	30	47	20	3		−1.127	0.260
49	I need support from management	42	42	16	–	23	60	17	–		−0.401	0.689
50	I need support from my colleagues in the nursing staff group	39	55	6	–	27	60	13	–		−1.384	0.166
51	I need support from my colleagues in the interdisciplinary team	39	51	10	–	30	60	10	–		−0.540	0.589
52	I need to believe that it has a positive effect on the patients	32	61	3	3	23	60	7	10		−1.611	0.107
53	I need to believe that it has a positive effect on me	16	74	7	3	13	60	17	10		−1.755	0.079
54	**I need to develop habits for working with a rehabilitative approach, which means incorporating it into my everyday life**	29	58	10	3	17	50	33	–		−2.170	**0.030**
55	**I need to develop plans for how to practically adopt a rehabilitative approach in my everyday work**	35	52	13	–	13	50	34	3		−2.977	**0.003**

Bold text indicates a statistically significant difference with a p-value less than 0.05

#### Competencies in relation to care and rehabilitation

The nursing staff assessed their knowledge of working with a rehabilitative approach (*p* = 0.013), knowing what patient involvement in rehabilitation means (*p* = 0.035), being sure about how to work with patients towards their goals for rehabilitation (*p* = 0.008) and their competence in performing tasks expected of an RN or NA in stroke rehabilitation to increase (*p* = 0.029). There were no changes in self-perceived competencies in respect to motivating patients to participate (*p* = 0.248) (Table 4).

#### Role and functions

We found an increase in knowledge of what was expected of them (*p* = 0.013). Before the educational programme10% stated that they did not know what was expected of them, whereas none stated this after. Furthermore, after the intervention, participants either agreed or totally agreed that they were so clear about their professional functions that they could communicate them to colleagues in the interdisciplinary team (*p* = 0.048).

#### Working with patient involvement

After the educational programme, the nursing staff stated that they totally agreed (10%), agreed (70%) or disagreed (20%) that the work in the unit was organised so that patients’ views and resources came into play, which contrasted with the responses before the educational programme (*p* = 0.023) (Table 4). The other 11 questions had no statistically significant changes.

#### Needs in order to get better

After the educational programme the nursing staff needed to a lesser degree than before to know more about why it was important to adopt a rehabilitative approach (*p* = 0.027) and expressed les need for education to know how to do so (*p* = 0.002). Furthermore, they professed less need for more practical skills (*p* = 0.030) after the educational programme (Table 4). Before the educational programme, they highlighted the need to develop the habit of working towards rehabilitation and planning for how this could be done practically; this result showed a statistically significant change in terms of a diminished need after the intervention (*P* = 0.003) (Table 4).

### Qualitative results

#### Physical capability

Participants articulated how working with patients in rehabilitation required experience, special abilities and skills. Physical capability was described as being related to basic and general nursing skills, but complex patients could require more than that:

*It may be that patients cannot move their extremities at all. It can be complex because they cannot speak or because they do not understand and cannot remember, or because they need complete help with all that is called ADL. (NA1)*

The nurse experienced physical capability as being linked to knowledge as a prerequisite physical skill. This includes knowledge of how, as a RN or NA, one is affected if they do not come to deploy one’s skills properly and how this could affect the patient. However, there was no direct expression of any effect on physical capability after participating in the educational programme.

#### Psychological capability

Nursing staff increased their knowledge and awareness of the importance of setting goals for patients. Goal-setting was described as a daily part of clinical practice. However, before the educational programme this was often not prioritised in a busy work day. The nursing staff became more aware of the need to establish rehabilitation goals after completing the programme. They also perceived a greater number of challenges associated with goal-setting, such as lack of documentation and continuity, and there was greater awareness of the benefits and relevance of goal-setting for patients: *We are working with the goals. I have greater insight into this now than I had previously (NA1).*

The nursing staff described having greater empathy for patients as well as greater understanding of their experiences of inpatient rehabilitation. This new knowledge inspired individual RNs or NAs to adjust their behaviour and actions in their daily practice:

*I was just wondering how much it really meant, how you [nursing staff] enter and are present. We often just act and think we have asked and involved the patient, but we may, in fact, only have mentioned it [what we are going to do] and then done it. (NA2)*

The observation exercise (Table 1) was described as increasing participants’ knowledge. It gave the observer insights into the patient’s perspective; what it meant to be under rehabilitation after suffering a stroke. The observation exercise was an eye opener to seeing the importance of nursing staff taking their time to involve the patients in activities of daily living (ADLs).

For some participants, it was surprising that their role and functions were described as therapeutically unspecific in the literature, but it helped to put things that they had been wondering about into perspective. Their understanding of their nursing role and functions increased after the intervention. They perceived the value of their contribution to patients’ physical training; for example, they recognised that they influenced patients’ rehabilitation as much as therapists did: *Well, I had the opinion (before) that training … was something that therapists did. It was an eye opener (NA3).*

The theory that was presented regarding their role and functions was new for most of the nursing staff, and they described not having experienced their role and functions in this light before, but that it made sense to them and provided the basis for increased capability.

*Well, now it is obvious after the project that we are a rehabilitation department. It became evident in the process that many of us, especially nurses and nursing assistants, are not* [as] *good at explaining or describing our professionalism and our role in rehabilitation as* [those in] *the other professional groups. (RN1)*

Greater awareness of one’s own role and functions was linked to increased motivation and ability to verbalise the contribution of the nursing staff to both the patients and the interdisciplinary team.

Participants described interdisciplinary collaboration as challenging; but increasing their knowledge of their own role and functions seemed to relieve some of these difficulties. They described having achieved a better understanding of a shared vision for the interdisciplinary team:

*I have come to understand the importance of working jointly to achieve goals that are set across disciplines*. […] *I think that I have got better at arguing for my own nursing versus their [the therapists’] training*. *Also, the things I can contribute with … are just as relevant as what they provide*, *because it is important for the patient. (RN2)*

In line with an increased understanding of their own role and functions, participants described gaining greater understanding of the concept of rehabilitation.

*The difference is that, for me, it has provided greater understanding and more knowledge of the specialism of stroke rehabilitation*. *For the patient, this means, of course, that there is now continuity in the way we work. I think all the things we’ve talked about* – *using each other and using everyone on the interdisciplinary team* – *that the physiotherapist and occupational therapist also use the same goals as us and work on them… and give time to the patients and train with patients – all those things help us to work using a rehabilitative approach. (NA 1)*

The nursing staff described that they were much more aware after the programme that little things they did with patients every day could be therapeutic in several ways, such as getting patients in and out of bed, helping them walk to the dining room, helping them get dressed etc.

#### Physical opportunities

The nursing staff described a lack of resources, especially relating to time and staffing levels. However, their participation in the educational programme and physical changes such as the new way of documenting the patients goals, emphasised by the programme, altered their understanding:

Besides directly influencing patient care, participants described lack of time and resources as affecting opportunities for professional advancement, as professional and mono- and interdisciplinary learning were not prioritised. Participation in the educational programme challenged their perceptions of professional advancement. Hence, the nursing staff came to view it not only as a personal professional gain, but also as having a direct impact on patient rehabilitation.

#### Social opportunity

The nursing staff lacked the mono- and interdisciplinary culture required to support the rehabilitative approach and the culture of goal-setting and systematic liaison with patients to work towards achieving their goals. A part of this culture could be documenting explicitly how and whether they worked rehabilitative or with goal-setting and what they expected more from their colleagues: *Personally, I think my colleagues document very little. There are some who do not write as much as I would like them to (RN 3).*

The nursing staff thought it was likely that a new common language and their enhanced professional role and functions would influence the work culture both internally among the nursing staff and in the interdisciplinary team.

#### Automatic motivation

The nursing staff expressed the wish to establish new work routines and habits, a process that some of them had already initiated:

*It’s not that I go around thinking about it for 8 hours, but there are times where there is a click in my head, and I think of it and try to do things differently.*[…]*. You have to repeat it over and over until it becomes automatic. But I’m getting better at it. (RN4)*

The newly acquired focus and knowledge from the educational programme were perceived to constitute the first step in establishing new behaviours. However, there were concerns that it would be difficult to continue to progress because of time pressure and lack of possibilities for ongoing development. The importance of management offering guidance and prioritising change was emphasised in that respect.

#### Reflective motivation

The value of having a stronger professional identity was expressed both implicitly and explicitly. Such a belief was also expressed as having the possibility of growing in their current circumstances: *I think it has been a really good offer in relation to strengthening professionalism and becoming aware of what is, in fact, necessary to care for these patients, doing straightforward things that we can easily benefit from like making changes within our existing framework* (RN5).

The nursing staff members had achieved a stronger professional identity and enhanced awareness of the possibilities of working with a rehabilitative approach:

*Because we are here 24 hours… and occupational and physiotherapists* [are] *not. We work* [all] *day, evening and night. There is actually training at night, because when patients need to get up from their bed and out into the hallway and go to the toilet. (RN6)*

This reflective motivation also related to making a difference for the patients; there was awareness that using a rehabilitative approach and working on goal-setting and patient involvement meant that nurses’ contributions could lead to important improvements in patients’ outcomes and experiences.

## Discussion

The results from both the qualitative and quantitative analyses, which in this discussion is merged, indicate that the educational programme shaped the target behaviours that we aimed to change by addressing the nursing staff’s capability, opportunity and motivation through an educational intervention, and hence had the potential to strengthen the nursing staff’s contribution to inpatient stroke rehabilitation.

The workshops mainly addressed cognitive functions. This was reflected in the interviews, where physical capability was considered not to have been directly changed after the intervention. However, in the quantitative data, the participants after the programme expressed less need for more practical skills to be able to work using a rehabilitative approach. This could be explained by the fact that the nursing staff in this study expressed the opinion that knowledge formed the basis of their clinical skills. A part of the Rehabilitation 24/7 educational programme included a role play exercise where the participants practiced communicating their professional functions to interdisciplinary collaborators (practiced skills). Appling this element as a practical skill exercise may be the reason why knowledge of how to collaborate and communicate with members of the interdisciplinary team stood out from the rest of the results, and it could indicate that this rehearsal positively influence the nursing staff’s learning. The fact that practical exercises had a positive influence on the learning is supported by the findings from an earlier study on nursing staff members’ beliefs, attitudes and actions in inpatient stroke rehabilitation. Here the participants stated that bedside training and peer-to-peer training was the best sources of learning [19]. This indicates the importance of integrating different approaches to learning.

Psychological capability seemed to increase overall in relation to the target behaviours. The quantitative results indicate a significant positive change from before to after the intervention with respect to working with patients towards achieving their goals. The qualitative results complemented this findings by illuminating how increased knowledge of the evidence behind goal-setting and why it is important in rehabilitation caused the nursing staff to prioritise goal-setting in their busy everyday practice. The quantitative and qualitative data also show that having a stronger professional identity is based on deeper insight into one’s own role and functions as well as increased understanding of the concept of rehabilitation. Mauk et al. [25] also found that education increased the nursing staff’s knowledge of nursing competencies in rehabilitation. Mauk et al. [25] argue that the specialism of rehabilitation requires rehabilitation-specific education both before and after graduation from nursing school. This was also suggested by Clark [9], who argues that stroke-specific education needs to be enhanced if nurses are to perceive more fully their rehabilitative role and enhance their rehabilitation skills. In previous research, nursing staff education is shown to have a positive effect on nurses’ self-perceived competence and job satisfaction and to increase quality in patient care [25]. However, only a few previous studies have addressed the issue of strengthening nurses’ contributions to inpatient stoke rehabilitation. Differences in content and duration of such educational initiatives make it difficult to compare our study to these prior studies [26–29]. Our previous study showed a high level of feasibility and acceptability of the educational programme among nursing staff [30]. According to Sidani [31], the acceptability of an intervention depends on the participants’ perception of the intervention. Hence, the high levels of acceptability and feasibility of the educational programme may have had a positive influence on the participants’ self-perceived outcome. However, recognizing that self-perceived effect has some degree of limitation when looking at effect, is important. First, a self-perceived effect does not necessarily report on the actual effect or change, but rather on the participants’ beliefs [13]. However, using a self-perceived outcome in a feasibility study can be a good indicator of the expected behaviour change. In an intervention aimed at professional behaviour change, intention, defined as the individual motivation concerning the performance of a given behaviour, is a good predictor for change [32]. According to Nielsen et al. [32], effect can only be expected if healthcare professionals have positive attitudes and good intentions.

Changes with respect to physical and social opportunities were seen at different levels. The quantitative results revealed no significant change regarding whether more time is needed to be better at working using a rehabilitative approach. However, the qualitative results are at variance with this as they offered descriptions of immediate changes in the structure of the unit after running the educational programme. Hence, increased understanding of the possibilities of integrating rehabilitation into basic daily care, thereby illustrating some differences in the perception of time. In a Q-methodology study whereby the participants sort a set of statements about nursing practice in inpatient stroke rehabilitation, Clarke and Holt [10] found that despite very real time and workload pressures, routinely integrated rehabilitation principles in care could be followed without extra time pressure. With respect to social opportunity, specifically having a supportive culture and norms for working with a rehabilitative approach, it was interesting from our study that more was expected from collaboration mono professional and among RNs and NAs and in the qualitative results were highlighted for fostering a good rehabilitative culture.

Overall, it appears that changes occurred in relation to all COM-B factors in our study. However the quantitative results relating to patient involvement did not show statistical significant changes in 11 out of 12 questions. This might reflect that the participant in the main part of these questions assess them self with a high level of agreement before the intervention. However the participants also stated that 83.9% felt stronger in working patient involving after participating which also was revealed in the qualitative analysis. This inconsistence in the results therefor calls for further investigation in order to strengthen the intervention.

While developing the educational programme, which was guided by the MRC framework and the BCW, we developed an implementation strategy by conducting a need assessment and following the steps proposed by Grol and Wensing for implementing change in health care [13]. This meant incorporating a multifaceted strategy for implementation during the testing period to support the educational programme, which allowed us to address several potential barriers. This strategy pointed to include, besides an educational intervention, other elements, such as feedback, reminders, etc. An educational intervention has been proven as effectual for increasing the knowledge of nursing staffs and is often a necessary step in implementing changes in practice [13]. However, the evidence for educational intervention is neither strong nor one-sided. When guided towards education as an element to achieve the desired behaviour change, considerations should be given to how. The Cochrane Effective and Organisation of Care Group (EPOC) distinguishe between different educational strategies, from e-learning to large-scale educational meetings [33]. In the current study, small-scale educational meetings were chosen. This decision was based on stronger evidence for effect [33] and didactic considerations and was deemed the most pragmatic in relation to the clinical practice. As such, we believe that it provided a strong foundation for a clinically relevant educational programme.

### Limitations

This study has several limitations. First, the tailoring of the intervention and the implementation strategy raise questions about the transferability of our results. We tried to address this issue by also conducting field observations in another context during the development phase to broaden both perspectives and context. Second, the questionnaire was only pre-tested on a small sample; therefore, its face validity could be questioned. Third, the study reports on results from a small sample; it is therefore important to interpret the statistical analyses cautiously. However, given the mixed-methods design, we interpreted the results in the light of both qualitative and quantitative data. Using a deductive approach in the qualitative analysis could mean a more narrowed insight into the perceived strengths and weaknesses of the program than an inductive approach might have been open for. However, applying a deductive approach using the COM-B factors as categories enhanced the possibility to draw clear lines back to the theoretical and empirical foundations of the intervention and thereby evaluate on these in line with the aim of the study. Fourth, a limitation of the study could be that the first author developed, delivered and evaluated the intervention. However, precautions were taken to limit any confounders; the interviews, for example, were performed by the second author, who were not directly involved in the development and delivery of the intervention, and analysis was performed collaboratively by the research team.

## Conclusions

The results of our study provide an understanding of the of the self-perceived outcome of the Rehabilitation 24/7 educational programme on nursing staff members’ behaviour. A mixed-methods approach provided us with in-depth knowledge of the changes in the nursing staff members’ behaviours following the intervention. These changes suggest that the different aspects of the Rehabilitation 24/7 programme strengthened the nursing staff’s knowledge and beliefs about rehabilitation as well as heightened their awareness of their own role and functions. Using a structured theory- and evidence-based approach guided by the BCW to address the COM-B factors and using a multifaceted implementation strategy appeared to enhance the effectiveness of the intervention.

In this study we created an educational intervention for nursing staff in practice. The intervention cost staffing hours during the test but was feasible and acceptable. The intervention was developed within theframework of an already existing context, which means it is possible to make behavioural changes for acollective staffing group within an already existing context. Hence this intervention should be considered as a way to enhance neuro-nursing.

The feasibility study of the rehabilitation educational programme seemed promising. Testing theintervention on a larger population in a controlled trial would be the natural next step. It could bebeneficial to do this as a multicentre study to increase the external generalisability. The present studywill contribute important knowledge about the process, content and structure. Before further investigation, future research should gain further understanding and testing of relevant outcome measurements both related to the nursing staff and patient outcomes.

## Abbreviations

ADL: Activities of daily living
COM-B: Capability Opportunity Motivation Behaviour
MRC: Medical Research Council
NA: Nurse Assistants
RN: Registered Nurse
